# Evaluation of a question generation approach using semantic web for supporting argumentation

**DOI:** 10.1007/s41039-015-0003-3

**Published:** 2015-06-23

**Authors:** Nguyen-Thinh Le, Niels Pinkwart

**Affiliations:** Department of Informatics, Humboldt-Universität zu Berlin, Unter den Linden 6, 10099 Berlin, Germany

**Keywords:** Question generation, WordNet, Argumentation

## Abstract

Discourse and argumentation are effective techniques for education not only in social domains but also in science domains. However, it is difficult for some teachers to stimulate an active discussion between students because several students might not be able to develop their arguments. This paper proposes to use WordNet as a semantic source in order to generate questions that are intended to stimulate students’ brainstorming and to help them develop arguments in a discussion session. In a study including 141 questions generated by human experts and 44 questions generated by a computer system, the following research questions have been investigated: Are system-generated questions understandable? Are they relevant to given discussion topics? Would they be useful for supporting students in developing new arguments? Are understandable and relevant system-generated questions predicted to be useful for students in order to develop new arguments? The evaluation showed that system-generated questions could not be distinguished from human-generated questions in the context of two discussion topics while the difference between system-generated and human-generated questions was noticed in the context of one discussion topic. In addition, the evaluation study showed that system-generated questions that are relevant to a discussion topic correlate moderately with questions that are predicted as useful for students in developing new arguments in the context of two discussion topics and understandable system-generated questions are rated as useful in the context of one specific discussion topic.

## Background

An argument is an artifact that is created to articulate and justify claims, explanations, or viewpoints, and argumentation is the process of generating these artifacts (Osborne et al. 2004; Sampson and Clark 2008). The ability to generate good arguments that involve evidence and theory to support or reject a claim or an explanation is an important component of inquiry learning (Sampson and Clark 2008; Duschl and Osborne 2002).

Questioning can be deployed to advance the argumentation ability of students, and teacher-initiated questions might stimulate the thinking process of students. Studies have reported that deploying questions can be effective for learning. With novice computer scientists, asking effective questions during the early phases of planning, a solution can support the students’ comprehension and decomposition of the problem at hand (Lane and VanLehn 2005). Asking targeted, specific questions is useful for revealing knowledge gaps with novices, who are often unable to articulate their questions (Tenenberg and Murphy 2005). Other researchers proposed to use questions to encourage students’ self-explanation. Questions of this type are referred to as explanation prompts and have demonstrated to be a promising instructional support feature (Berthold et al. 2011) and highly beneficial for learning (Chi et al. 1994). Questions can not only be used as a teaching technique by teachers; Yu and Liu (2008) reported that requesting students to pose questions by themselves during the learning process helps students develop both cognitive and metacognitive strategies.

### Research questions

The goal we pursue in our research is to generate questions automatically in order to support students in developing their own arguments for a given discussion topic so that they could improve their argumentation ability and would be more active in a discussion session. As the first step on the way to achieve this goal, in this paper, we investigate whether WordNet (Miller 1995), a lexical database for English, is an appropriate source for generating questions automatically. For this purpose, we will investigate the following research questions:

1. Are questions that are generated using WordNet as understandable as human-generated questions?
2. Are questions that are generated using WordNet as relevant to a given discussion topic as human-generated questions?
3. Are questions that are generated using WordNet perceived as useful as human-generated questions?
4. Are understandable and relevant system-generated questions predicted to be useful for students in order to develop new arguments?

### State of the art of using questions in technology-enhanced learning

In this section, educational applications of automatic question generation are reviewed and classified. This paper extends the four classes of educational applications of question generation proposed in Le et al. (2014) with a new class: prompts for education.

The first class includes systems that pose prompts to students and have proven to be effective in supporting cognitive and meta-cognitive learning strategies (Glogger et al. 2009; Wong et al. 2002). Prompts are hints or questions that induce productive learning processes. Prompting assumes that learners already know certain learning strategies, but that they are not able to apply them appropriately. Prompts are supposed to overcome the deficiency of applying learning strategies, that is, a student’s lack of application of a helpful strategy that is already in a student’s repertoire (Glogger et al. 2009; Flavell 1978). Prompts can also be used to support journal writing. Writing learning journals, students are instructed to write down a text in which they reflect on the previous classes’ learning contents and their learning process. Berthold et al. (2007) found that cognitive prompts or a combination of cognitive and meta-cognitive prompts elicited significantly more corresponding learning strategies compared to no prompts or just meta-cognitive prompts. Schwonke et al. (2006) also reported benefits of deploying adaptive cognitive and meta-cognitive prompts to help students revise learning journals. Nückles et al. (2009) compared the usefulness of different sets of prompts for writing journals and reported that participants, who received cognitive and meta-cognitive prompts including hints on planning or remedial strategies, outperformed the participants in the other conditions (no prompts, only using cognitive prompts, only meta-cognitive prompts, cognitive and just monitoring prompts as meta-cognitive prompts).

The second class of applications of automatic generated questions includes systems that are intended to help students acquire knowledge or skills. Kunichika et al. (2001) proposed an approach to extracting syntactic and semantic information from an original text and questions are constructed based on the extracted information. The authors reported that 80 % of the automatically generated questions were considered as appropriate for novices learning English by experts. Aiming at improving reading skills of students, Mostow and his research group (for instance, Mostow et al. 2008; Mostow et al. 2013) developed an automated reading tutor which generates questions automatically for enhancing the student’s comprehension of text reading. Mostow and Chen (2009) investigated how to generate self-questioning instruction automatically on the basis of statements about mental states (e.g., belief, intention, supposition, and emotion) in narrative texts. The reading tutor has been evaluated with respect to the acceptability of menu choices (grammatical, appropriate, and semantically distinct), the acceptability of generated questions, and the accuracy of feedback. Mostow and Chen (2009) reported that only 35.6 % of generated questions could be rated as acceptable. In the same class of educational applications of question generation, Liu and colleagues (Liu et al. 2012) introduced a system (G-Asks) for improving students’ writing skills (e.g., citing sources to support arguments, presenting the evidence in a persuasive manner). The approach implemented in this system consists of three stages. First, citations in an essay written by the student are extracted, parsed, and simplified. Then, in the second stage, the citation category (opinion, result, aim of study, system, method, and application) is identified for each citation candidate. In the final stage, an appropriate question is generated using pre-defined question templates. Evaluation studies have shown that the system could generate questions as useful as human supervisors and significantly outperformed human peers and generic questions in most quality measures after filtering out questions with grammatical and semantic errors (Liu et al. 2012).

The third class of educational applications of question generation aims at assessing the knowledge of students. Heilman and Smith (2009) developed an approach to generating questions for assessing students’ acquisition of factual knowledge from reading materials. The authors developed general-purpose rules to transform declarative sentences into questions. The approach includes an algorithm to extract simplified statements from appositives, subordinate clauses, and other constructions in complex sentences of reading texts. Evaluation studies have been conducted to assess the quality and precision of automatically generated questions using Wikipedia and news articles. The authors reported that the acceptability of top-ranked WH questions is around 40–50 %. Furthermore, K-12 teachers created factual questions by selecting and revising suggestions from the system with less effort than by writing questions on their own (Heilman 2011). One common form for assessing student’s factual knowledge is the use of multiple-choice tests. Mitkov and colleagues (Mitkov et al. 2006) developed a computer-aided environment for generating multiple-choice test items. The authors deployed various natural language processing techniques (shallow parsing, automatic term extraction, sentence transformation, and computing of semantic distance). In addition, the authors exploited WordNet, which provides language resources for generating distractors for multiple-choice questions. In addition to generating test items automatically, the system provides the user the option to post-process the test items. The authors reported that the time required for generating questions including manual correction was less than for manually creating questions alone (Mitkov et al. 2006). Also with the purpose of assessing students’ knowledge, Brown and colleagues (Brown et al. 2005) developed the system REAP which is intended to provide students with texts to read according to their individual reading levels. The system chooses text documents which include 95 % of words that are known to the student while the remaining 5 % of words are new to the student and need to be learned. After reading the text, the student’s understanding is assessed. The system generates different types of questions including word bank and multiple-choice questions. In contrast to Mitkov and colleagues who used WordNet to generate distractors, Brown et al. (2005) used WordNet to generate different types of questions (definition, synonym, antonym, hyperonym, hyponym, and cloze questions). Experimental results have been reported that with automatically generated questions, students achieved a measure of vocabulary skill that is comparable to performance on independently developed human-generated questions. Another form of assessing student’s knowledge is to rely on fill-in-the-blank questions. Hoshino and Nakagawa (2005) proposed to deploy standard classification methods to decide the position of the gap in a fill-in-the-blank item. Sumita et al. (2005) developed fill-in-the-blank questions by replacing verbs with gaps in an input sentence. Possible distractors are retrieved from a thesaurus by choosing the same Part of Speech (e.g., noun, verb, adjective) and similar word frequency in a tagged corpus. A new sentence is created by placing a distractor in the gap position in the original sentence and is then used as the input for a search on the Internet. If the sentence is found on the Internet, the distractor is considered invalid. Here, participants who took a test consisting of automatically generated items achieved scores that highly correlated with their scores in the Test of English for International Communication (TOEIC).

The fourth class of educational applications of question generation includes systems that are able to provide tutorial dialogues. Olney and colleagues (Olney et al. 2012) presented a method for generating questions for tutorial dialogue. This involves automatically extracting concept maps from textbooks in the domain of Biology. This approach does not deal with the input text on a sentence-by-sentence basis only. Rather, various global measures (based on frequency measures and comparison with an external ontology) are applied to extract an optimal concept map from the textbook. Person and Graesser (2002) developed an intelligent tutoring system that improves students’ knowledge in the areas of computer literacy and Newtonian physics using an animated agent. Each topic contains a focal question, a set of good answers, and a set of anticipated bad answers (misconceptions). The system initiates a session by asking a focal question about a topic and the student is expected to write an answer containing 5–10 sentences. Initially, the system used a set of predefined hints or prompts to elicit the correct and complete answer. Graesser and colleagues (Graesser et al. 2008) reported that with respect to learning effectiveness, the system had a positive impact on learning with effect sizes of 0.8 standard deviation units compared with other appropriate conditions. Lane and VanLehn (2005) developed PROPL, a tutor which helps students build a natural-language style pseudo-code solution to a given problem. The system initiates four types of questions: 1) identifying a programming goal, 2) describing a schema for attaining this goal, 3) suggesting pseudo-code steps that achieve the goal, and 4) placing the steps within the pseudo-code. Through conversations, the system tries to remediate a student’s errors and misconceptions. If the student’s answer is not ideal (i.e., it cannot be understood or interpreted as correct by the system), sub-dialogues are initiated with the goal of soliciting a better answer. PROPL has been evaluated with the programming languages Java and C and it has been reported that students who used this system were frequently better at creating algorithms for programming problems and demonstrated fewer errors in their implementation (Lane and VanLehn 2005).

In contrast to traditional approaches to generating questions using text as input and deploying various natural language processing techniques for creating questions, the fifth class of educational applications of question generation exploits linked open data that are a part of the semantic web (Heath and Bizer 2011) for generating questions. Jouault and Seta (2013, 2014) proposed to generate semantics-based questions by querying information from the large linked open data sources DBpedia (http://dbpedia.org/) and Freebase (https://www.freebase.com/) to facilitate learners’ self-directed learning. Using this system, students in self-directed learning are asked to build a timeline of events of a history period with causal relationships between these events given an initial document. The student develops a concept map containing a chronology by selecting concepts and relationships between concepts from the given initial Wikipedia document to deepen their understanding. While the student creates the concept map, the system also generates its own concept map by referring to semantic information from DBpedia and Freebase. The system’s concept map is updated with every modification of the student’s one and enriched with related concepts that can be queried from both linked open data sources. Thus, the system’s concept map always contains more concepts than the student’s map. Using these related concepts and their relationships, the system generates questions for the student to lead to a deeper understanding without forcing to follow a fixed path of learning.

Five classes of existing educational applications of automatic question generation have been reviewed. The fifth class of educational applications, which make use of the semantic web for generating questions, needs more research. At present, to our best knowledge, just the work of Jouault and Seta (2013, 2014) falls in this research direction. In light of this research gap, this paper proposes to use WordNet in order to generate questions that aim at stimulating the brainstorming of students during the process of argumentation. WordNet (cf. “Methods” section) has been decided to be used as a semantic source for generating questions because it is a rich lexical database that is able to provide hyponyms (related concepts) to a queried concept. We hypothesize that hyponyms could be used to generate questions that are related to a given discussion topic.

Although the question generation approach presented in this paper and the work of Jouault and Seta are intended to help students deepen their understanding in a learning/discussion topic by working with generated questions, our approach is different from the work of Jouault and Seta in two points: 1) With respect to the technical issue, while Jouault and Seta adopted ontology and linked open data techniques to eliminate the difficulty of the natural language understanding problem in the learning domain (in this case, the history domain), this paper suggests an approach to deploy natural language techniques (e.g., a natural language parser) in order to extract important concepts from a discussion topic and using WordNet to query related concepts that are relevant for discussion; 2) With respect to learning goals, Jouault and Seta proposed to use automatic generated questions for enhancing students’ knowledge in history whereas our approach focuses on helping students develop new arguments for the argumentation process.

## Methods

### Question generation using WordNet

In this section, we describe conceptually how questions can be generated in our approach. A more detailed technical description of the approach is presented in Le et al. (2014b). In order to illustrate the question generation approach proposed in this paper, we will use the following discussion topic that can be given to students in a discussion session:

The catastrophe at the Fukushima power plant in Japan has shocked the world. After this accident, the Japanese and German governments announced that they are going to stop producing nuclear energy. Should we stop producing nuclear energy and develop renewable energy instead?

From the discussion topic, we note that the following noun phrases can serve as starting points to generate questions: *catastrophe*, *Fukushima power plant*, *nuclear energy*, *renewable energy*. This step is described in more details in the following subsection.

#### Analyzing text structure and identifying key concepts

**Fig. 1:**
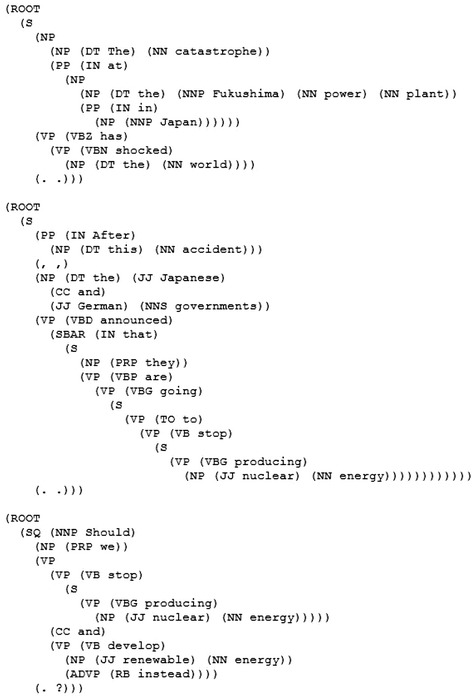
Parse tree of the discussion topic. This parse tree has been generated using the Stanford parser: http://nlp.stanford.edu:8080/parser/index.jsp

In order to automatically recognize key concepts of a discussion topic, a natural language parser is used to analyze the grammatical structure of a sentence into its constituents. The language parser analyzes a text and identifies the category of each constituent (for instance: determiner, noun, or verb). This parsing process results in a parse tree. Since nouns and noun phrases can be used as key concepts in a discussion topic, we select from the parse tree of the parsed discussion text only constituents which are tagged as nouns (NN) or noun phrases (NP) (cf. Fig. 1). Since the present implementation of our approach is not able to determine which concept is more important than another one. Thus, the system proposed here uses all extracted key concepts that are marked as NN or NP in the resulted parse tree.

#### Question generation using noun phrases in a discussion topic

Using the extracted key concepts, we are ready to generate questions. The next issue that needs to be addressed is to determine the types of questions to be generated. According to Wilen (1991), there exist more than 21 classification systems for classroom questions (e.g., Bloom (1956), Otero and Graesser (2001), Schreiber (1967), Pate and Bremer (1967), and Graesser and Person (1994)). While Bloom’s taxonomy is widely used for classroom teaching (Arias de Sanchez 2013), the question taxonomy for tutoring proposed by Graesser and Person (1994) is specialized for one-on-one tutoring. This taxonomy consists of 16 question categories: verification, disjunctive, concept completion, example, feature specification, quantification, definition, comparison, interpretation, causal antecedent, causal consequence, goal orientation, instrumental/procedural, enablement, expectation, and judgmental. The first 4 categories are classified as simple/shallow, 5–8 as intermediate, and 9–16 as complex/deep questions. We apply this question taxonomy to define appropriate question templates for generating questions, because it is more fine-grained than Bloom’s taxonomy, and as stated, has been designed for one-on-one settings (cf. Table 1). Using defined question templates, we are able to replace the placeholder X by nouns and noun phrases extracted from a discussion topic. For example, the following question templates are filled with the noun phrase “nuclear energy” and result in some questions.

**Table 1:** Question templates proposed for question generation

Type	Question
Definition	What is <X>?
	What do you have in mind when you think about <X>?
	What does <X> remind you of?
Feature/property	What are the properties of <X>?
	What are the (opposite)-problems of <X>?
	What features does <X> have?
Example	What is an example of <X>?
Verification	Is there any problem with the arguments about <X>?
Judgment	What do you like when you think of or hear about <X>?
Interpretation	How can <X> be used today?
Expectation	How will <X> be in the future, based on the way it is now?
Quantification	How many *sub-topics* ^a^ did your partners talk about?
	Which *sub-topics* do your partners focus on?
Concept comparison	What is the difference or relations between these *sub-topics*?

^a^For the question categories “Quantification” and “Concept comparison”, we do not use a placeholder. Instead, *sub-topics* indicate different concepts being discussed by the discussion partners or provided by a hyponym set

- *What does <X> remind you of?*
- *What are the properties of <X>?*
- *What is an example of <X>?*

#### Question generation using related concepts in WordNet

Semantics-based question generation approaches use a source of semantic information which is related to the topic being discussed. Since in this paper we focus on using semantic information available on the Internet for generating questions, the source of “semantic information” we look for is on the semantic web. For example, Wikipedia (https://www.wikipedia.org/) provides descriptions of concepts. While Wikipedia might contain incorrect information due to its contribution mechanism, one of the advantages of Wikipedia is that the description of many concepts is available in many different languages. If we want to develop a question generation for different languages, Wikipedia might be an appropriate source. WordNet (Miller 1995) also provides a source of semantic information which can be related to a discussion topic. WordNet is an online lexical reference system for English. Each noun, verb, or adjective represents a lexical concept. A concept is represented as a synonym set (called synset), i.e., the set of words that share the same meaning. Between two nominal synsets, WordNet provides semantic relations. The hyponym relation represents a concept specialization. For example, for the concept “energy”, WordNet provides a list of direct hyponyms which are directly related to the concept being searched and represent specializations: “activation energy”, “alternative energy”, “atomic energy”, “binding energy”, “chemical energy”, and more. In addition, a synset can contain example sentences, which can be used for generating questions. For example, for a concept of “energy” into WordNet, an example sentence like “energy can take a wide variety of forms” for this concept is available. One of the advantages of WordNet is that it provides accurate information (e.g., hyponyms) and grammatically correct example sentences.

Placeholders in question templates (Table 1) can be filled with appropriate hyponym values for generating questions. For example, the noun “energy” exists in the discussion topic, and after extracting this noun as a key concept, it can be used as input for WordNet that provides several hyponyms, including “activation energy”. The following question templates can be used to generate questions of the question category “Definition” (see Table 2).

**Table 2:** An example of question template for the question class “Definition”

Type	Question template	Question
Definition	What is <X>?	What is activation energy?
	What do you have in mind when you think about <X>?	What do you have in mind when you think about activation energy?
	What does <X> remind you of?	What does activation energy remind you of?

### Evaluation

The goal of the evaluation is to determine whether automatically generated questions are of as high quality as human-generated questions. That is, we want to know whether an automatically generated question can be identified by human raters and how they rate the quality of system-generated questions as compared to human-generated questions.

In the first evaluation phase, we invited eight experts from the research communities of argumentation and question/problem generation to manually create questions. We gave them the following three discussion topics and asked them to create questions which can be used to support students in developing arguments. Since the eight experts work in USA, Europe, and Asia, we chose discussion domains with international relevance which had been in the news recently. For this study, we chose the domains of energy and economy. Each discussion topic consisted of two sentences and an initial discussion question. This kind of construction for discussion topics was intended because discussion participants and human experts should have enough “materials” for thinking about a specific problem. If a discussion topic was too short (e.g., only a sentence or a discussion question), this might make it difficult for discussion participants to initiate a discussion or for human experts to think of questions to be generated:

Topic 1: *The catastrophe at the Fukushima power plant in Japan has shocked the world. After this accident, the Japanese and German governments announced that they are going to stop producing nuclear energy. Should we stop producing nuclear energy and develop renewable energy instead?*

Topic 2: *Recently, although the International Monetary Fund announced that growth in most advanced and emerging economies was accelerating as expected. Nevertheless, deflation fears occur and increase in Europe and the US. Should we have fear of deflation?*

Topic 3: *“In recent years, the European Central Bank (ECB) responded to Europe's debt crisis by flooding banks with cheap money…ECB President has reduced the main interest rate to its lowest level in history, taking it from 0.5 to 0.25 percent” (Kwasniewski* 2013). *How should we invest our money?*

From our eight experts, we received 54 questions for topic 1, 47 questions for topic 2, and 40 questions for topic 3.

For each discussion topic, the system generated several hundred questions (e.g., 844 questions for topic 1), because from each discussion topic several key concepts were extracted, and each key concept was extended with a set of hyponyms queried from WordNet. For each key concept and each hyponym, fourteen questions have been generated based on the question templates in Table 1. Since the set of generated questions was too big for expert evaluation, in the second evaluation phase, we selected a small amount of automatic generated questions randomly, so that the proportion between the automatic generated questions and the human-generated questions was about 1:3. There were two reasons for this proportion. First, in case the proportion between automatically generated questions and human-generated questions is too high, then it could influence the real “picture” of human-generated questions. Second, we needed to make a trade-off between having enough (both human-generated and system-generated) questions for evaluation and considering a moderate workload for human raters. The proportion of automatic generated questions and of human-generated questions is in Table 3.

**Table 3:** Number of questions generated by human experts and by the system

	Topic 1	Topic 2	Topic 3
	No. of questions	No. of questions	No. of questions
Human-generated	54	47	40
System-generated	16	15	13
Total	70	62	53

Then, we mixed human-generated questions with automatic generated questions and asked human raters to identify whether each question from the mixed set of questions had been generated by the system or by a human expert. For topic 1, we had three raters, and for each of the last two topics, we could only get two raters. Note that these human raters were not the same human experts who generated questions. Also, they did not know about the proportion between human-generated questions and system-generated questions.

## Results

### Evaluation of human perception

First, we evaluated the soundness of system-generated questions. For this purpose, we asked human raters to answer the following question: *Is that an automatic system-generated question (Yes/No)?* We use the balanced *F*-score to evaluate and to analyze the ratings of humans. The *F*-score is calculated based on precision and recall using the following formula:

$$ F=\frac{2\ast \mathrm{precision}\ast \mathrm{recall}}{\mathrm{precision}+\mathrm{recall}} $$

The precision for a class is the number of true positives (i.e., the number of system-generated questions correctly labeled as belonging to the positive class) divided by the total number of elements labeled as belonging to the positive class, while the recall for a class is the number of true positives divided by the total number of elements that actually belong to the positive class. If the *F*-score is high, it shows that the system-generated questions and the human-generated questions are easy to distinguish. Otherwise, a low *F*-score indicates that it is difficult for human raters to distinguish between system-generated and human-generated questions.

Table 4 summarizes the *F*-scores of each human rater. It shows that for topic 1, it was difficult for rater 1 (*F* = 0.33) and moderately difficult for rater 2 (*F* = 0.51) to distinguish the authorship of questions. The kappa value (0.086) indicates a low agreement between two raters—which means that even if each of the graders correctly classified some questions, their ratings would not be consistent with each other. With respect to topic 2, the *F*-score of both raters is moderate (0.5 and 0.52). The Kappa value for their agreement was 0.233, which can be considered as fair. This shows that for topic 2, it was easier to distinguish between human-generated and system-generated questions than in the context of topic 1. With respect to topic 3, for both raters, it was relatively difficult to identify the authorship of the questions (*F*-score is between 0.40 and 0.44) and the agreement between the raters was fair (0.263).

**Table 4:** *F*-score of two raters for the authorship of questions

	Rater 1			Rater 2		
	*F*-score	Precision	Recall	*F*-score	Precision	Recall
Topic 1 (inter-rater agreement Kappa = 0.086)	0.33	0.75	0.21	0.51	0.81	0.37
Topic 2 (inter-rater agreement Kappa = 0.233)	0.50	0.87	0.35	0.52	1.00	0.35
Topic 3 (inter-rater agreement Kappa = 0.263)	0.40	0.77	0.27	0.44	0.92	0.29

Interestingly, in the context of topic 3, one question “*What is cheap money?*” was generated by a human expert and by the system identically. This question was assumed *by both human raters as a system-generated question*. Thus, this question was not included in the statistical evaluation for topic 3.

In summary, we have learned that for all raters it was not easy to identify system-generated questions from the set of mixed questions. This indicates that system-generated questions are sound as human-generated questions. The agreement between raters was slight or fair. This strengthens the indication that it was difficult for human raters to distinguish between system-generated and human-generated questions.

### Evaluation of question quality

The goal of the following evaluation is to empirically investigate the first three research questions specified in the “Background” section: 1) Are the system-generated questions understandable? 2) Are they relevant to the given discussion topic? 3) Would they be useful for supporting students in developing arguments?

The first three research questions were also given literally to human raters who were asked to rate the mixed set of questions using the scale from one to three scores (1: least, 2: middle, 3: most). First, we investigate these research questions in the context of each specific discussion topic, then we normalize the evaluation result for each topic and investigate these research questions in general.

In the context of topic 1, Table 5 shows that the mean of understandability for human-generated questions (2.28) is a little higher than of system-generated questions (2.19). However, their difference is statistically not significant. With respect to the relevance of the questions to the given discussion topic, the mean of the score for human-generated questions (2.14) is also higher than of the system-generated questions (1.96) and their difference is not significant. However, in the context of the usefulness of questions for supporting students in developing arguments: the mean of human-generated questions (2.12) is higher than of system-generated questions (1.69) and the difference is significant. In summary, in the context of topic 1, the first and the second research questions can be confirmed while the third one must be rejected.

**Table 5:** Quality of questions for topic 1

	Understandability	Relevance	Usefulness
	Mean (s.d.)	Mean (s.d.)	Mean (s.d.)
System-GQ	2.19 (0.89)	1.96 (0.87)	1.69 (0.69)
Human-GQ	2.28 (0.80)	2.14 (0.86)	2.12 (0.87)
Difference	*t* = 0.67	*t* = 1.25	*t* = 0.39
Significance	*p* = 0.51 (not significant)	*p* = 0.21 (not significant)	*p* = 0.0009 (significant)

Analyzing the system-generated questions in the context of topic 1, we learned that there was no question that was rated with score 1 (i.e., least understandable, least relevant, and least useful) on average. The list of system-generated questions that have the rating score of 1.33 on average with respect to “Usefulness” follows:

- *What do you have in mind when you think about tsunami?*
- *What do you like when you think of/about catastrophe?*
- *What does Fukushima remind you of?*
- *What does power plant remind you of?*
- *What features does catastrophe have?*

The low usefulness of these questions might be attributed to the fact that these questions are very general and have little relation to the question in the discussion topic 1 (“*Should we stop producing nuclear energy and develop renewable energy instead*”). If the questions were more specific, for example, “*What does the catastrophe at the Fukushima power plant in Japan remind you of*?”, this could be more useful.

In the context of topic 2, Table 6 shows that the human-generated questions are statistically significant better than system-generated questions on all three criteria: understandability (*t* = 3.01), relevance (*t* = 3.93), and usefulness (*t* = 3.29). Thus, the research hypothesis that system-generated questions are understandable, relevant to a given discussion topic, and useful for developing new arguments as human-generated questions cannot be confirmed in the context of topic 2.

**Table 6:** Quality of questions for topic 2

	Understandability	Relevance	Usefulness
	Mean (s.d.)	Mean (s.d.)	Mean (s.d.)
System-GQ	2.40 (0.77)	1.83 (0.7)	1.87 (0.82)
Human-GQ	2.76 (0.48)	2.43 (0.73)	2.37 (0.70)
Difference	*t* = 3.01	*t* = 3.93	*t* = 3.29
Significance	*p* = 0.0031 (significant)	*p* = 0.0001 (significant)	*p* = 0.0013 (significant)

We investigated the system-generated questions which had least mean score, i.e., the rating mean score over the raters is 1. Table 7 shows that the questions that have the lowest mean score contain “non-meaningful” nouns/noun phrases (“fear of deflation”, “international monetary”, “state capitalism”, and “deflation”) and these nouns/noun phrases are not in accordance with the meaning of the other constituents of a question. That is, the constituents of a question were in contradiction, for example: “How can *deflation* be *used* today?” It is not common for us that deflation can be “used” (unless we are economy experts). The other problem with these questions is that these “non-meaningful” nouns/noun phrases are extracted from the discussion topic (e.g., “fear of deflation”, “international monetary”) and from the hyponym set provided by WordNet (“state capitalism”). This is a limitation of the question generation approach presented in this paper. In the current version, the system is not implemented with a mechanism to identify meaningful noun phrases from the set of noun phrases that are extracted from a discussion topic and from the hyponym set of WordNet.

**Table 7:** System-generated questions that have the lowest mean score in the context of topic 2

Least understandable question	Are there any problem with arguments about fear of deflation?
Least relevant question	How can international monetary be used today?
Least useful questions	Are there any problem with arguments about fear of deflation?
	Are there any problem with the arguments about state capitalism?
	How can deflation be used today?

Similar to topic 1, in the context of topic 3, Table 8 shows that human-generated questions are better, but not significantly, than system-generated questions on all three criteria. This confirms that our research questions can be answered with “Yes” on the criteria “Understandability”, “Relevance”, and “Usefulness”.

**Table 8:** Quality of questions for topic 3

	Understandability	Relevance	Usefulness
	Mean (s.d.)	Mean (s.d.)	Mean (s.d.)
System-GQ	2.21 (0.72)	1.92 (0.88)	1.71 (0.69)
Human-GQ	2.27 (0.80)	2.21 (0.78)	2.04 (0.76)
Difference	*t* = 0.33	*t* = 1.54	*t* = 1.89
Significance	*p* = 0.7397 (not significant)	*p* = 0.1272 (not significant)	*p* = 0.0613 (not significant)

We analyze the system-generated questions with the lowest scores. We identified one least understandable, two least relevant, and one least useful question(s) (Table 9). The least understandable question can be attributed to the noun phrase “*(opposite-) problems*” that is generated by the system using a pre-specified question template. The question could be more understandable if it were constructed like this: “*How could problems of the central bank be stopped?*” Thus, the pre-specified question template should be optimized accordingly. The problems with the two least relevant questions can be explained by the noun phrases “*ECB president*” and “*central bank*” that are not as relevant as other noun phrases “*debt crisis*” and “*cheap money*” in topic 3. Again, the problem here is to determine the most important noun phrases in a discussion topic before applying question templates for constructing questions. The least useful question “*What features does ECB president have?*” was also rated as least relevant. In In the “Discussion” section, we will discuss about this issue and approaches to determining important concepts.

**Table 9:** System-generated questions that have least mean score in the context of topic 3

Least understandable question	How could (opposite-) problems of central bank be stopped?
Least relevant questions	What features does ECB president have?
	What is an example of central bank?
Least useful question	What features does ECB president have?

The question “*What is cheap money?*” that was generated identically by a human expert and by the system was rated by both human raters as very understandable. However, with respect to the criteria “Relevance” and “Usefulness”, there was disagreement between raters as Table 10 shows. Low kappa values of agreement between the human raters can be attributed to different strategies of distinguishing between system-generated questions and human-generated questions. Some human raters informed us about the different criteria they used to identify system-generated questions: 1) a question is superficial with regard to a given discussion topic, 2) a question is similar to another one in the mixed set of questions, 3) a question that expects a factual answer and is intuitive (e.g., “What features does ECB president have?”), 4) a question that contains unknown information (e.g., “How will those policies affect those outcomes/stakeholders?”), 5) human-generated questions may have typo/syntax errors, while system-generated questions are error-free.

**Table 10:** Ratings for a specific question that was generated identically by a human expert and the system

Understandability			Relevance			Usefulness		
Rater 1	Rater 2	Mean	Rater 1	Rater 2	Mean	Rater 1	Rater 2	Mean
3	3	3	3	1	2	2	1	1.5

Overall, when considering the quality of system-generated questions over all three topics, we can learn from Table 11 that there is no significant difference between the human-generated and system-generated questions, i.e., the system-generated questions are as understandable as human-generated questions. That means, the first research question can be answered in the affirmative. However, with respect to the relevance of questions to the given discussion topics and to the usefulness of the questions, the human-generated questions are significantly better, and thus, the second and the third research questions can be answered in the negative.

**Table 11:** Quality of questions over all three topics

	Understandability	Relevance	Usefulness
	Mean (s.d.)	Mean (s.d.)	Mean (s.d.)
System-GQ	2.25 (0.82)	1.91 (0.82)	1.75 (0.73)
Human-GQ	2.41 (0.76)	2.23 (0.81)	2.17 (0.78)
Difference	*t* = 1.78	*t* = 3.49	*t* = 4.94
Significance	*p* = 0.0758 (not significant)	*p* = 0.0005 (significant)	*p* < 0.00001 (significant)

### Correlation between understandability, relevance, and usefulness

In this section, we investigate the fourth research question: Are understandable and relevant system-generated questions also useful for students?

In the context of topic 1 (cf. Table 12), we can note that system-generated questions that are relevant to discussion topic 1 have a strong positive correlation with the criterion “Usefulness” (*r* = 0.76). A similar tendency can be found for human-generated questions (*r* = 0.81). Both correlation values are significant. However, the understandable system-generated questions are weakly correlated with the criterion of usefulness (*r* = 0.31), whereas for human-generated questions the correlation between the criteria understandability and usefulness is higher (*r* = 0.57).

**Table 12:** Correlation between understandability, relevance and usefulness in the context of topic 1

	Correlation between understandability and usefulness	Correlation between relevance and usefulness
System-GQ	0.31 (weak relationship)	0.76 (strong positive)
	*p* = 0.03 (significant)	*p* < 0.00001 (significant)
Human-GQ	0.57 (moderate positive)	0.81 (strong positive)
	*p* < 0.00001 (significant)	*p* < 0.00001 (significant)

In contrast to topic 1, in the context of discussion topic 2 (cf. Table 13), we can learn that for both system-generated questions and human-generated questions, the correlation between the criteria “Relevance” and “Usefulness” is weak (*r* = 0.14–0.17, not significant). Yet, correlation values show that understandable questions (either system-generated or human-generated) are moderately correlated with the criterion of being useful questions (*r* = 0.52–0.53) and these correlation values are significant.

**Table 13:** Correlation between understandability, relevance, and usefulness in the context of topic 2

	Correlation between understandability and usefulness	Correlation between relevance and usefulness
System-GQ	0.52 (moderate positive)	0.14 (weak relationship)
	*p* = 0.003 (significant)	*p* = 0.46 (not significant)
Human-GQ	0.53 (moderate positive)	0.17 (weak relationship)
	*p* < 0.00001 (significant)	*p* = 0.10 (not significant)

In the context of topic 3 (cf. Table 14), for both classes of questions (human-generated and system-generated), the correlation between understandability and usefulness is positive (*r* = 0.39–0.43). However, it indicates a weak relationship between understandable questions and useful questions. The correlation between the relevance of a question and its usefulness (*r* = 0.53–0.62) is moderately positive and means there is a tendency that relevant questions will be useful for students. Note, except the correlation coefficient between the criteria understandability and usefulness for system-generated questions, all other correlation values are significant.

**Table 14:** Correlation between understandability, relevance, and usefulness in the context of topic 3

	Correlation between understandability and usefulness	Correlation between relevance and usefulness
System-GQ	0.39 (weak relationship)	0.53 (moderate positive)
	(*p* = 0.06, not significant)	(*p* = 0.0077, significant)
Human-GQ	0.43 (weak relationship)	0.62 (moderate positive)
	(*p* = 8.9E-05, significant)	(*p* < 0.00001, significant)

In summary, the fourth research question, whether understandable and relevant questions would be useful for students, can apparently confirmed in most cases. Understandable questions (both system-generated and human-generated questions) are significantly correlated with useful questions, except the system-generated questions for topic 3. Relevant questions (both system-generated and human-generated questions) are significantly correlated with useful questions, except for topic 2.

## Discussion

The question generation approach has been evaluated using three discussion topics from the domains of energy (topic 1) and economy (topics 2 and 3). Each topic was presented by two sentences that describe the problem of a topic, followed by a discussion question. With two discussion domains, we still cannot conclude about the coverage of scope of discussion domains that can be supported by the question generation system using WordNet. However, the results of the evaluation study give us some information about the quality of system-generated questions. In the context of topic 1, the human-generated questions were not significantly better than system-generated questions over three criteria “Understandability” and “Relevance” (however, with respect to “Usefulness”, human-generated questions were more useful). In the context of topic 3, the difference between human-generated questions and system-generated questions was not significant over three criteria. Only in the context of topic 2, which is about increasing fear of deflation in Europe and US, the difference between human-generated and system-generated questions was statistically significant, i.e., the quality of human-generated questions was better than of system-generated questions. Of course, the effectivity of our approach relies on the set of hyponyms provided by WordNet and on the accuracy of the algorithm that extracts nouns/noun phrases from a discussion topic.

In the current implementation of the system, the algorithm for extracting nouns/noun phrases from a discussion topic has the limitation that it is not able to rank the importance of a noun/noun phrase. In order to determine the relevance of a concept, several effective approaches have been devised in the area of information retrieval, e.g., document frequency (Joho and Sanderson 2007) and term frequency-inverted document frequency (Baeza-Yates and Ribeiro-Neto 1999). Document frequency is calculated by the number of documents which contain a specific term in the corpus of documents. Term frequency is used as a numerical statistic to determine how important a word is to a document in a corpus or how important a word is to a corpus. Usually, the factor “inverse document frequency” is incorporated in the term frequency algorithm to diminish the weight of terms that occur very frequently in the document corpus and increases the weight of terms that occur rarely. These approaches could be investigated to be included in the algorithm for extracting relevant concepts from the discussion topic.

With respect to the selected amount of system-generated questions for the evaluation study, we selected only a small number of system-generated questions among a huge number of generated questions (over 800 for topic 1) for evaluation without having clear selection criteria. The small number of selected system-generated questions and the ratio 1:3 between system-generated questions and human-generated questions might not reflect fully the quality of system-generated questions. We might think of increasing this ratio. Yet, possibly too many system-generated questions might bias human graders—this needs to be investigated.

## Conclusions

This paper presented a question generation approach using WordNet for supporting students during argumentation processes. The approach extracts important concepts from a discussion topic and query hyponyms of these concepts from WordNet. Questions are constructed by either using important concepts from a given discussion topic or using hyponyms of the extracted concepts.

Although the evaluation results show that system-generated questions were as sound as human-generated questions in two discussion topics, the question generation approach presented in this paper certainly still has some limitations. First, it generates too many questions for a discussion topic. Second, the algorithm for extracting relevant concepts is not yet able to determine the grade of importance for each noun/noun phrases. These two issues are our short-term future work.

As long-term future work, we intend to use system-generated questions and human-generated questions of highest quality to test whether they are actually useful for students in the argumentation process. After that, we intend to identify and model characteristics of useful questions for argumentation purposes. Using this model, appropriate question templates will be defined for question generation.
